# Contributing factors to the total fertility rate declining trend in the Middle East and North Africa: a systemic review

**DOI:** 10.1186/s41043-021-00239-w

**Published:** 2021-03-25

**Authors:** Aboulghasem Pourreza, Ahmad Sadeghi, Mostafa Amini-Rarani, Rahim Khodayari-Zarnaq, Hasan Jafari

**Affiliations:** 1 Department of Health Management and Economics, School of Public Health, Tehran University of Medical Sciences, Tehran, Iran; 2 Department of Public Health, Esfarayen Faculty of Medical Sciences, Esfarayen, Iran; 3 Social Determinants of Health Research Center, Isfahan University of Medical Sciences, Isfahan, Iran; 4 Tabriz Health Services Management Research Centre, Iranian Center of Excellence in Health Management, School of Management and Medical Informatics, Tabriz University of Medical Sciences, Tabriz, Iran; 5 Health Policy and Management Research Center, Department of Health Care Management, School of Public Health, Shahid Sadoughi University of Medical Sciences, Yazd, Iran

**Keywords:** Total fertility rate, Systematic review, Middle East and North Africa, Determinant factors

## Abstract

**Background:**

The total fertility rate (TFR) in the Middle East and North Africa has experienced a declining trend in recent years. Accordingly, the present study was conducted to provide a clear picture of the most critical factors affecting the TFR decline in this region.

**Methods:**

This study was a systematic review between the years 2000 and 2016. The different databases like Cochrane, PubMed, Scopus, and Science Direct and the Google Scholar search engine were used. At first, 270 articles and then 18 articles were selected and meticulously read for the final analysis.

**Results:**

The results indicated a declining trend in the TFR in the Middle East and North Africa, as in other parts of the world. Regarding the causes of this declining trend, several factors were identified and categorized into five main factors of health care-related, cultural, economic, social, and political.

**Conclusions:**

While taking advantage of the experiences, it is necessary to identify the five main factors and their related issues and hence consider them in the population policy-making.

## Introduction

Today, the population is one of the most critical economic, social, and infrastructural components of every planning and policy-making process worldwide. Changes in population can play a significant role in the economic and social development of each society [1]. One of the factors affecting population changes is the rate of fertility, and the most important indicator for estimating it is the total fertility rate (TFR) defined as the average number of children per a woman would have during her fertility life. A generation can survive if each woman gives birth to at least two children, but there are men and women who do not marry or, if married, do not have any children for some reason. Hence, each couple should give birth to an average of 2.1 children, so that the population remains constant [2].

In recent years, especially in the last decade of the twentieth century, most countries have experienced a decline in the TFR. Global TFR has decreased over the past seven decades, and it has been experienced a constant decrease since 1950. TFR was 4.97 in 1950, 4.40 in 1970, 3.18 in 1990, 2.72 in 2000, and 2.31 in 2019 [3]. The TFR was almost five children per woman in the early 1950s, and then it decreased to 4.5 in the early 1970s and less than three in the 1990s. Of course, there have been significant differences in the TFR decline between various countries [4]. Developing countries, like other countries in the world, have experienced a rapid decline in the TFR in recent decades [5]. Therefore, except for a limited number of African countries, the TFR has been declining throughout the world. Meanwhile, the fastest decline in the TFR has been reported in Asia, North Africa, and Latin America where a relatively rapid socioeconomic development is also observed [6]. Among these regions is the Middle East and North Africa (MENA) region, where the TFR has declined from almost seven children in 1950 to 2.5 children in 2019 [3].

The decline in TFR was first observed in Lebanon and then in other countries such as Egypt, the Islamic Republic of Iran, and Turkey. It is worth mentioning that the last three countries were among the first countries that officially supported family planning programs. In the 1960s, these governments started implementing a series of family planning programs to improve national health conditions and guarantee a low population growth as part of their national development policies [7]. Among the MENA region countries, Iran, Lebanon, Tunisia, and Turkey have reached a TFR below the replacement level [8]. In recent years, population aging and low TFR and their adverse effects have given rise to various economic and political considerations in the MENA region countries and led to changes in the population policies of some countries, including Iran and Turkey [9].

Accordingly, population-based studies identifying factors affecting the TFR decline in the MENA region have become more critical. Due to high sociocultural similarities between countries included in the MENA region, conducting systematic reviews on factors affecting the TFR decline in this region can help develop evidence-based population policies. No systematic review has been conducted so far to address the causes of TFR decline in the MENA region. Accordingly, the present systematic review was conducted to fill this gap.

## Methods

The present study was a systematic review of studies conducted about the factors affecting the TFR decline in the MENA region. The study’s population consisted of all research articles, published in the English language between the years 2000 and 2016. It should be mentioned that the MENA region was selected for the present analysis due to high cultural, social, and economical similarities and also epidemiological transitions among included countries.

### Searching strategy

**Fig. 1:**
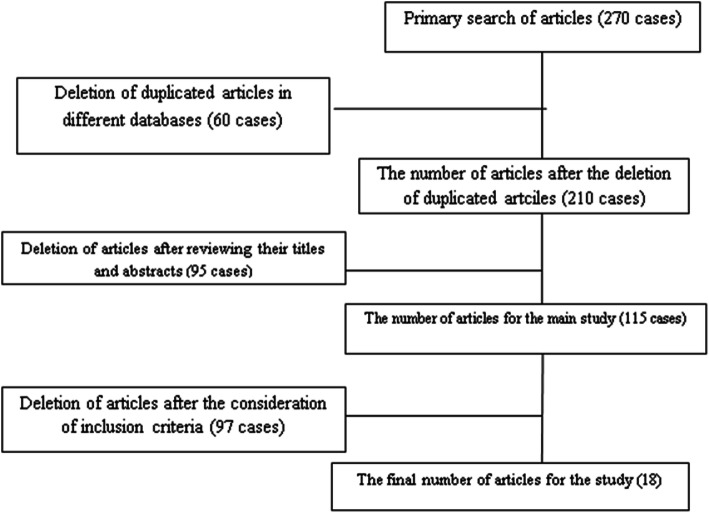
The process of research selection for the identification of TFR decline factors

Using the *PubMed*, *Springer*, *Cochrane*, *Scopus*, *Science Direct*, and ISI *Web of Science* databases and the Google Scholar search engine, the keywords “population decline,” “population decrease,” “population fall,” “Middle East and North Africa,” “TFR/total fertility rate determinants,” and “Muslim countries” were looked for in the title and abstract sections of the available articles. Moreover, the reference lists of the found articles were reviewed to find more relevant studies. Thus, 270 research articles on the TFR decline in the MENA region were initially found. Then, similar or duplicated articles were screened out, and 210 entered the next stage of the analysis. At that point, irrelevant articles were removed from the data set after reviewing their titles, abstract sections, and main bodies. Finally, 18 research articles, found to be entirely in line with the present study’s objectives, were entered the study, meticulously read, and examined systematically. Figure 1 shows how the articles were included in/excluded from the present analysis. The final set of articles was also evaluated by a checklist covering the necessary data, including the titles, the journal titles, the authors’ names, the time and the studies’ objectives. The inclusion criteria included being written in the English language, being published after the year 2000, and being conducted on the identification of factors associated with TFR decline in the MENA region.

## Results

After searching, screening, and evaluating the examined studies, the 18 studies in terms of the TFR decline in the MENA region were identified. The summary features of these studies are shown in Table 1. Two of these studies were gathered data from 74 [8, 10] and 67 [8, 11] countries around the world including MENA countries. One of these studies identified the causes of the fertility rate in six UNICEF regions [12]. Six studies were conducted on 44 Muslim-majority countries [14], 22 countries and areas of the Arab region [23], global Muslim population [20], Arab world [21], and Muslim world [22] which included MENA region countries in terms of fertility decline causes. Other nine studies [13, 15–19, 24–26] specifically were focused on the MENA region. As shown in Table 2, the findings were classified into five main factors including health care-related, cultural, economic, social, and political factors affecting the TFR decline in the MENA region. Also, issues corresponding to each main factor are presented in Table 2. The analysis showed that health care-related factors, such as reduced rate of maternal/infant mortality, increased rate of contraceptive use, and improved maternal/infant health services, are significant contributing factors to the TFR decline in the MENA region.

**Table 1:** A summary of the characteristics of included articles concerning the causes of TFR decline in the MENA region

Author/authors	Title	Year
United Nations, Population Division [10]	Fertility levels and trends in countries with intermediate levels of fertility,	2002
United Nations, Population Division [11]	Views and policies concerning population growth and fertility among governments in intermediate-fertility countries	2002
Norville, C., Gomez, R., and Brown, R. L .[12]	Some causes of fertility rates movements	2003
Yousef M [13]	Development, growth and policy reform in the Middle East and North Africa since 1950	2004
Karim M [14]	Socio-economic development, population policies, and fertility decline in Muslim countries	2004
Matthiessen P [15]	The demography of the Middle East and North Africa in a global context,	2005
Roudi-Fahimi F, Kent MM [16]	Challenges and opportunities: the population of the Middle East and North Africa	2007
Clawson P [17]	Demography in the Middle East: population growth slowing, Women’s situation unresolved	2009
Mirkin B [7]	Population levels, trends and policies in the Arab region: challenges and opportunities	2010
Hosseini H [18]	Comparative study of fertility transition and population ageing in four selected countries in the MENA region	2010
Akhtar, Sh., Reinnika, R., Jorgensen, S., Maeda, A [19]	Meeting the challenges of health transition in the Middle East and North Africa	2010
Lugo, L., Cooperman, A., O’Connell, E., Stencel, S [20]	The future of the global Muslim population, projections for 2010-2030	2011
Crane, K., Simon, S., Martini, J [21]	Future challenges for the Arab world, the implications of demographic and economic trends	2011
Eberstadt N, Shah A [22]	Fertility decline in Muslim world: a veritable change, still curiously unnoticed	2011
Mirkin B [23]	Arab Spring: demographics in a region in transition	2013
Salehi-Isfahani D [24]	The role of the family in social integration in the Middle East and North Africa	2013
Bommes, M., Fassmann, H., Sievers, W [25]	Migration from the Middle East and North Africa to Europe, past developments, current status and future potentials	2014
Iqbal F, Kiendrebeogo Y [26]	The reduction of child mortality in the Middle East and North Africa: a success story	2014

**Table 2:** Main factors contributing to the decline of TFR in the MENA region

Main factor	Issues
Health care-related	Improved primary care services and integration of family planning programs [12, 16, 18, 22, 23], greater confidence of parents in the survival of their children [21], reduced rate of infant/child/maternal mortality [11, 17, 22, 24–26], increased rate of modern contraceptive use [7, 10, 11, 17, 19–23], promoted maternal-child health services [17], increased beds/hospitals relative to population size [15], increased men’s participation in reproductive/sexual health practices [23]
Cultural	Changes in women’s attitudes towards employment [12], weakening of traditional values and norms of MENA societies concerning parenting and child-raising, and strengthening of their tendencies to the norms of Western cultures [14, 16, 21], increased women’s tendency to get married at older ages [10, 21, 23], changes in people’s beliefs about marriage and parenting [18], increased tendency to form smaller families [21, 23]
Economic	High costs of marriage and child-raising [12, 13, 21, 23], inflation [18], housing and employment problems [11, 13, 23], economic development and improved living standards [20], increased countries’ per capita income [15, 24], youth’s economic expectations, increased GDP per capita [20]
Social	Increased urbanization [7, 10, 12, 14, 17, 20–22], increased educational levels (especially of women) [7, 10–13, 15–17, 19–24], women’s empowerment [7, 17], increased rate of migration (especially to foreign countries) [21], reduced rate of early marriage [23]
Political	Governments’ direct support policies for family planning [11, 13–15, 20, 21, 23], abolishment of restrictions to the realization of women’s rights (e.g., revision of divorce laws) increased legal age of marriage [21, 23], the establishment of restrictions for polygamy [21], attempts to reduce people’s tendency to have male children [23]

Changes in women’s attitude towards employment and early marriages, reduced interest in family formation among younger people, weakening of traditional values and norms of societies concerning parenting and child-raising, and strengthening of their tendencies to the norms of western cultures were found to be the most important cultural factors causing the TFR decline in the MENA region.

The third group of contributing factors to the declining trend of TFR in the MENA region was the economic factors, including increased costs of child-raising, inflation, increased rates of women’s employment, housing and employment problems for younger people, and youth’s economic expectations.

Furthermore, various articles emphasized the role of social factors in the TFR decline. Among these social factors, increased urbanization, increased educational level (especially of women and girls), women’s empowerment, high rate of migration, and decline in early marriage were extracted. Lastly, several studies identified political factors affecting the TFR decline in the MENA region which include governments’ direct and formal support and policies for reducing population growth and developing family planning programs, and indirect policies for improving the general education level and eliminating restrictions to the realization of women’s rights.

## Discussion

Results of the present systematic review of articles conducted on the main factors causing the TFR decline in the MENA region indicated that this decline is associated with various health care-related, cultural, economic, social, and political factors.

One of the influential main factors is health care-related factors. In a study on the global TFR decline, Basu (2002) showed that the rate of fertility remains high when the rate of infant mortality is high, because parents are not sure about the survival of their children [27]. Bongaarts and Casterline (2012) emphasized the critical role of increased use of contraceptives in the TFR decline in Sub-Saharan Africa. Lack of knowledge about the available contraception methods and their providers, low-quality and limited access to family planning services, the costs of contraception and concerns about its impacts on women’s health, lack of husbands’ or other family members’ willingness to use contraceptives, and finally concerns about the social and ethical acceptability of contraception can be mentioned as barriers to the use of contraceptives [28]. Hirschman (2001) believed that increased use of contraceptives is the most important factor in the TFR decline in Southeast Asian countries [29]. Sleebos (2003) mentioned the increasing rate of contraceptives use as one of the important causes of TFR decline among member countries of the Organization of Economic Co-operation and Development (OECD) [30]. Similarly, Westley and colleagues (2010) and Westoff and colleagues (2013) reported increasing rates of the use of health and family planning technologies among residents of Asia and Sub-Saharan Africa [31, 32]. Therefore, in line with results of the present systematic review, results of other studies emphasized the importance of health care-related factors in the TFR decline in different parts of the world.

The next main factor corresponding to the TFR decline in the MENA region is cultural ones. Sleebos (2003) stated that, in a post-capitalist and post-materialist era, people prefer to stay single longer, or if they marry, they will not stay in their relationships for a long time; hence, childbirths will be postponed. Residents of the MENA region are somehow influenced by the Western culture. Therefore, what was stated by Sleebos can be considered relevant to people living there [30].

Addio and Ercole (2005) believed that one of the most important causes of the TFR decline among member countries of the OECD is young women’s changing attitudes towards the traditional roles of women in the family and the society. Hirschman (2001) emphasized the vital role of religion, as a cultural attribute, in Southeast Asian countries. He believed that the TFR of Malaysian women is higher than that of women in other parts of the world due to the impacts of Islamic culture on them [29]. Nonetheless, the present study’s results were not in line with Hirschman’s findings regarding the impact of religious values on the TFR decline. In fact, despite Islam’s emphasis on marriage, the TFR has declined to the replacement level, or even below it, in many Muslim countries in the MENA region, such as Iran, Tunisia, Lebanon, and Turkey. Accordingly, despite the importance of Islamic values, environmental, political, social, and demographic characteristics of each region may eventually determine the TFR.

Another identified main factor to the declining trend of TFR in the MENA region is economic factors. Westley and colleagues (2010) pointed out that one of the important factors in the TFR decline in the East Asian region is the decreasing number of marriages, which is due to the high costs of marriage. Moreover, the decision whether or not to have children is profoundly affected by the costs of child-rearing, which include the costs of parenting and losing job opportunities [31]. Martine and colleagues (2013) showed that changes in couples’ demand for having children originate from changes in their income level and relatively high costs of child-raising [33]. Sleebos (2003) recognized two scenarios regarding the impact of economic conditions on the TFR of member countries of the OECD: at the time of economic prosperity, more women enter the labor market, so that they have less time for child-raising. On the other hand, at the time of economic downturn, financial uncertainties and lower family income levels may again lead to the decline of TFR [30].

Social factor was another main factor for the decline in the TFR in the MENA region. Sleebos (2003) showed that factors, such as women’s higher levels of education and their tendency to postpone marriage until the end of their studies, can be considered essential causes of the TFR decline in member countries of the OECD [30]. Likewise, Addio and Ercole (2005) indicated that women with higher education and income levels tend to have lower numbers of children [34]. This result was also confirmed in the present study. In Hirschman’s study on the fertility transition in Southeast Asia, factors such as increased levels of education and women’s and non-agricultural employment were identified as influential factors in the TFR decline [29].

From Westley and colleagues’ point of view, increased women’s educational levels and rate of employment along with improved living standards have led to a decline in the TFR [31]. Similarly, Westoff and colleagues believed that the TFR decline in Sub-Saharan countries is due to people’s increased educational levels and their exposure to public media, especially TV [32]. Basu (2002) believed that, in some African countries and parts of Asia (e.g., India and Pakistan), women’s and girl’s education has strengthened their connections to public media and changed their aspirations [27]. On the contrary, Sleebos (2003) found higher rates of fertility among women with higher levels of education. These different results may be explained by considering the different evolutionary paths of developed and developing countries and their specific socioeconomic conditions [30]. Regarding the impact of urbanization—as a social factor—on the TFR decline, Bongaarts and Casterline [28] stated that people in societies with lower levels of urbanization (e.g., African societies) tend to have more children because they help their parents in farming practices and are sources of security as they age. Westoff and colleagues (2003) considered the increased rate of urbanization as one of the causes of people’s changing attitudes towards the ideal number of children [32]. Hirschman (2001) also believed that urbanization is an influential factor in the TFR decline in Southeast Asia. Sleebos (2003) proposed that the shifts from traditional, agriculture-based societies to industrial ones are among the causes of TFR decline among member countries of the OECD [30]. In general, the results of many studies were consistent with the results of the present analysis regarding the impact of social factors on the TFR decline. Therefore, it can be concluded that social factors, such as increased educational level and a higher rate of urbanization, have affected the TFR decline in the MENA region, like other parts of the world.

Finally, political factor was identified as another influential main factor in terms of TFR decline in the MENA region. Hirschman [29] assumed that governments’ support for family planning programs is the main reason for the increased rate of contraceptives use in Southeast Asia [29]. This finding was consistent with the results of the present systematic review. Hirschman also emphasized the importance of governments’ indirect policies for declining the TFR in the MENA region. For example, the governments’ indirect policies played a more effective role in India and China than in Malaysia [29].

## Conclusions

The results indicated a declining trend in the TFR in the MENA region, as in other parts of the world. In general, studies on the causes of TFR decline in the MENA region have reached a consensus about the impacts of the mentioned five factors (i.e., health care-related, social, cultural, economic, and political) on the TFR decline in the MENA region. Accordingly, to find solutions to overcome the TFR decline below the replacement level, while taking advantage of the experiences of all countries, it is necessary to identify the five factors and consider them in evidence-based decision-making processes and population policies.

## Data Availability

All data generated or analyzed during this study are included in this published article.
